# Incidence of Gastric Neoplasms Arising from Autoimmune Metaplastic Atrophic Gastritis: A Systematic Review and Case Reports

**DOI:** 10.3390/jcm12031062

**Published:** 2023-01-30

**Authors:** Chuyan Chen, Yi Yang, Peng Li, Haiyi Hu

**Affiliations:** Department of Gastroenterology, Beijing Friendship Hospital, Capital Medical University, National Clinical Research Center for Digestive Disease, Beijing Digestive Disease Center, Beijing Key Laboratory for Precancerous Lesion of Digestive Disease, Beijing 100050, China

**Keywords:** autoimmune metaplastic atrophic gastritis, pernicious anemia, gastric cancer, dysplasia, neuroendocrine tumor, gastric hyperplastic polyps

## Abstract

Autoimmune metaplastic atrophic gastritis (AMAG) is associated with an increased risk of gastric neoplasms. This study aimed to systematically analyze the incidence rate of gastric cancer (GC), low-grade dysplasia (LGD) and type-1 gastric neuroendocrine tumor (gNETs) development in AMAG adults. Studies on AMAG patients reporting the incidence of gastric neoplasms was identified through a systematic search in PUBMED and EMBASE. Study quality was assessed using the Joanna Briggs Institute quality assessment tool. Incidence rates of GC, LGD and type-1 gNETs were examined by meta-analysis. Thirteen studies met eligibility criteria. Incidence rate of gastric cancer calculated from the pooled data was 0.14% per person-year in both single-center studies and national registration studies. Meta-analysis showed a relative risk of 11.05 (95% CI: 6.39–19.11) for gastric cancer development in AMAG patients. The calculated pooled gastric LGD and type-1 gNETs incidence rates were 0.52% and 0.83% per person-year, respectively. As for experience from our center, we presented three distinctive cases of gastric neoplasm arising from the background of AMAG. This study underscores the potential for malignant transformation of precancerous lesions and reiterates the importance of careful esophagogastroduodenoscopy screening.

## 1. Introduction

Autoimmune metaplastic atrophic gastritis (AMAG) is an immune-mediated chronic inflammatory disease characterized by progressive damage of the oxyntic glands and destruction of parietal cells, leading to advanced mucosal atrophy, intestinal metaplasia and hypergastrinemia [1,2]. The loss of parietal cells also causes reduced or absent production of the intrinsic factor, which is responsible for transportation of vitamin B12 to the terminal ileum for absorption, resulting in deficiency of vitamin B12 and development of pernicious anemia (PA) [3].

As a consequence of chronic inflammation, AMAG patients are linked to increased risk of gastric neoplastic changes. Before understanding of the biology of AMAG, PA has been used as a synonym for AMAG, and the risk of cancer development in patients with PA has been reported in literature. A systematic review published in 2013 showed that the pooled gastric cancer (GC) incidence rate was 0.27% per person-year in PA patients, which reached a relative risk of 6.8 compared with general population [4]. However, PA is only part of the AMAG clinical spectrum and may be considered as a late stage of AMAG [2]. Therefore, it is necessary to further explore the risk of gastric cancer in AMAG patients. Moreover, with the popularization of esophagogastroduodenoscopy (EGD), more and more precancerous lesions such as dysplasia have also been discovered and reported in studies. We also aim to analyze the incidence rate of low-grade dysplasia (LGD) in this study.

AMAG also predisposes patients to develop type-1 gastric neuroendocrine tumors (gNETs). Advanced oxyntic mucosa damage results in impaired gastric acid secretion and hypergastrinemia, which stimulates the growth of enterochromaffin-like (ECL) cells, leading to ECL cell hyperplasia, dysplasia and type-1 gNETs [5]. In this study, we also reviewed literature to estimate the incidence rate of type-1 gNETs in AMAG patients.

Our study aims to provide an overview of incidences of different gastric lesions in AMAG patients. Additionally, we present three distinctive cases of gastric neoplasm arising from the background of AMAG.

## 2. Materials and Methods

### 2.1. Search Strategy

This systematic review was performed according to the Preferred Reporting Items for Systematic Reviews and Meta-Analyses (PRISMA) 2020 Statement [6], and the protocol was registered on INPLASY (INPLASY2022120021). Articles providing information on AMAG and gastric neoplasms were identified through a systematic search in PUBMED and EMBASE by using various combinations of the following terms (Table S1): autoimmune gastritis, atrophic gastritis, Type A gastritis, pernicious anemia, macrocytic anemia, vitamin B12 deficiency, cobalamin deficiency, intrinsic factor deficiency, gastric cancer, gastric adenocarcinoma, stomach cancer, gastric neoplasm, gastric carcinoma, gastric tumor, gastric neuroendocrine tumor, gastric carcinoid, gastric dysplasia and gastric polyp. Search of the database was conducted for articles published up to 24 September 2022. Additionally, references of retrieved articles were screened for eligibility.

### 2.2. Study Selection and Eligibility Criteria

All the records were imported to EndNote X8 (Clarivate Analytics, Toronto, ON, Canada) and duplicate records were removed manually. Observational studies including patients with AMAG or PA and which reported the numbers of gastric neoplastic lesions identified during a specified follow-up period were eligible for inclusion in this systematic review. Studies which met the below conditions were excluded: (Ⅰ) they were case reports, reviews, letters, or editorials; (Ⅱ) they were not original data or they were repeat publications; (Ⅲ) no follow-up data were available. Two reviewers (CY.C. and Y.Y.) independently selected studies based on their titles and abstracts. Disagreements were resolved by another investigator (HY.H.).

### 2.3. Data Extraction and Quality Assessment

The following information was extracted independently by two reviewers (CY.C. and Y.Y.): title, first author, country of study location, year of publication, sources of selection of participants, study design, criteria for diagnosis of AMAG, numbers of patients investigated, gender distribution, age of patients, duration of follow-up period (years), whether follow-up was active or not, methods of follow-up and numbers of cases with gastric neoplastic lesions (including GC, gastric LGD and type-1 gNETs).

All included studies were assessed for quality by two reviewers (CY.C and Y.Y.) using the Joanna Briggs Institute (JBI) quality assessment tool [7]. Studies were assessed by rating list of 10 questions, including adequacy of sampling, description and data analysis. For each question that was answered “Yes”, one point was received. The risk of bias for each study was divided into three categories: low risk (7–10 points), moderate risk (4–6 points) and high risk (less than 4 points). Any disagreements were discussed and resolved.

### 2.4. Statistical Analysis

Incidence rate of different gastric lesions was calculated as the ratio between the number of new gastric lesions detected over the follow-up period and the number of person-years observed. All data analyses were performed using STATA 15.0 software. The chi-squared test was used to assess statistical heterogeneity. When I^2^ < 50%, it was considered as ‘no obvious heterogeneity’ and the fixed-effects model was applied. Otherwise, the random-effects model was used. Statistical significance was determined by a *p*-value of <0.05. The pooled incidence rate of gastric cancer, gastric LGD and type-1 gNETs per person-year and its 95% confidence intervals were calculated. Subgroup analysis was performed by study design (single-center or national registration studies) and study population (AMAG or PA) to analyze incidence rate of gastric cancer. Based on data from studies following active surveillance, the annual incidence rates of gastric cancer in AMAG patients were compared with the annual gastric cancer incidence rates reported by GLOBOCAN 2020 [8] (both genders, aged over 40 years, continent corresponding to study location) to estimate the relative risk (RR) of gastric cancer in AMAG patients.

## 3. Results

### 3.1. Search Results

**Figure 1:**
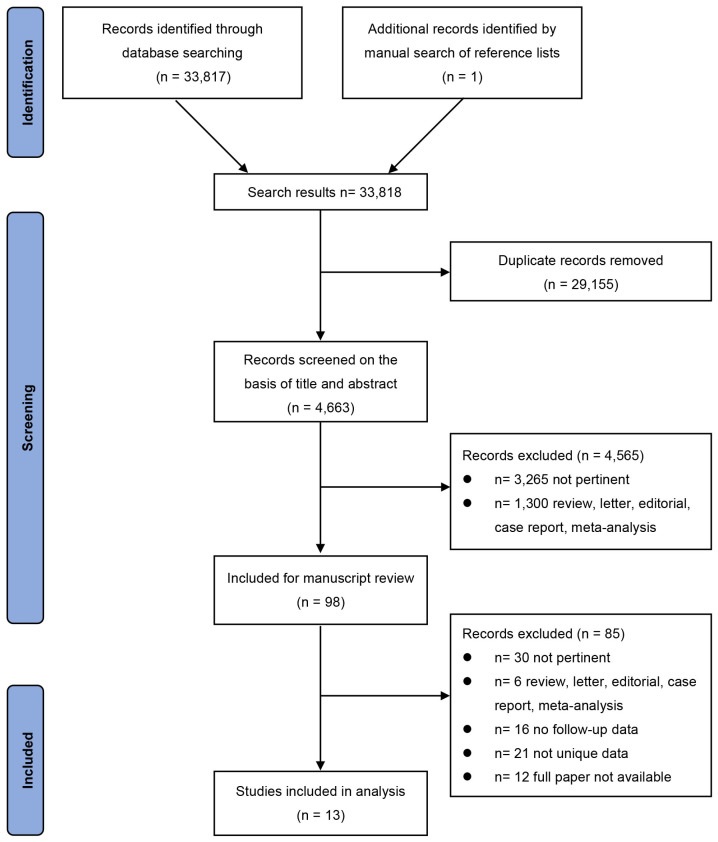
Flow chart of study selection.

The database searches identified a total of 33,817 potentially relevant articles. Another article was added after screening the references of selected papers. Of these articles, 4663 were unique. A total of 98 articles were retrieved and reviewed for full text after screening their titles and abstracts. Finally, the systematic review included 13 eligible articles [9,10,11,12,13,14,15,16,17,18,19,20,21]. The detailed procedure for literature selection is shown in Figure 1.

### 3.2. Quality Assessment

As evaluated by the JBI tool, 12 studies (92.3%) showed a low risk of bias, and 1 study showed a moderate risk of bias. The final quality scores of the included studies are represented in Table S2.

### 3.3. Characteristics of Studies

Ultimately, our analysis included 13 articles, mostly from European countries (69.2%). Other studies came from countries such as China and the United States. Three studies were national registration studies, and the remaining were single-center studies or regional studies. The sample size of single-center studies varied from 59 to 270. Twelve out of thirteen studies had a prevalence in female gender, ranging from 57% to 80.4%. The USA Veterans hospitalization study included only male patients. The median age of patients across all studies was 65.5 years. The nomenclature and diagnostic criteria of AMAG evolved over time, reflecting the progressive knowledge accumulated about the disease. Early studies mostly focused on the incidence of gastric cancer in PA patients, whereas recent studies tried to describe the incidence of different gastric lesions in AMAG patients and focused more on precancerous lesions. Detailed descriptions of the included studies are presented in Table 1.

### 3.4. Identification of Gastric Neoplasms

**Figure 2:**
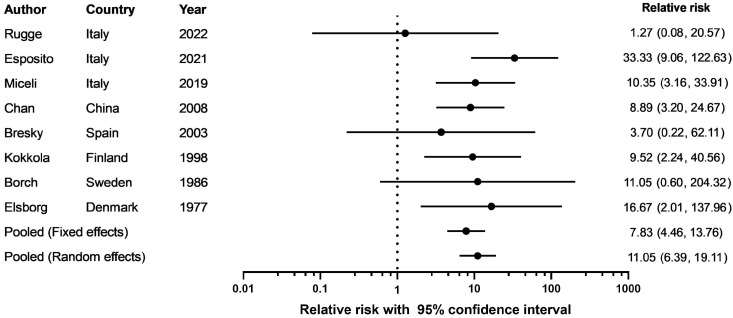
Gastric cancer relative risk of the eight studies with active follow-up in AMAG patients [9,10,11,13,14,15,16,18].

In single-center or regional studies, the incidence rate of gastric cancer per person-year was 0.14% based on the pooled data (95% CI: 0.01–0.35, *p* = 0.0128, I^2^ = 57.1%). Similarly, meta-analysis of national registration studies also showed a pooled gastric cancer incidence rate per person-year of 0.14% (95% CI: 0.09–0.19, *p* = 0.0019, I^2^ = 84.0%). The pooled gastric cancer incidence rate in studies constrained to AMAG was 0.28% (95% CI: 0.01–1.05, *p* = 0.0015, I^2^ = 80.5%), while the pooled incidence rate in PA patients was 0.10% (95% CI: 0.01–0.25, *p* = 0.3548, I^2^ = 9.56%, fixed-effect model). As shown in Figure 2, the overall gastric cancer relative risk in AMAG was 11.05 (95% CI: 6.39–19.11).

**Figure 3:**
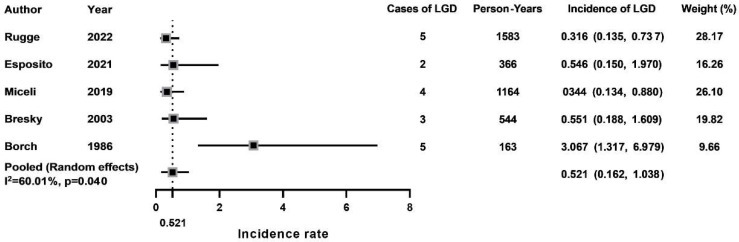
Gastric LGD incidence rates in AMAG patients [9,10,11,14,16].

Five studies were included to calculate the pooled incidence rates of LGD per person-year. Additionally, due to high statistical heterogeneity (*p* = 0.040, I^2^ = 60.01%), a random-effect model was adopted. According to Figure 3, the pooled incidence rate of LGD in AMAG patients was 0.52% (95% CI: 0.16–1.04) per person-year.

**Figure 4:**
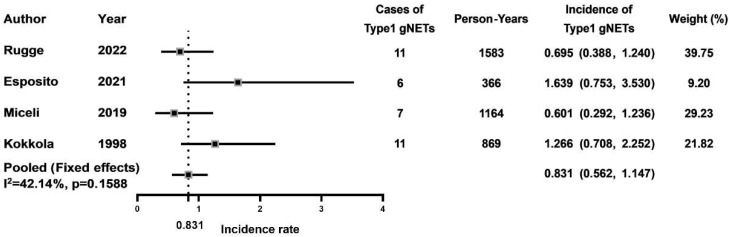
Incidence rates of type-1 gNETs in AMAG patients [9,10,11,15].

Four studies were included to calculate the pooled incidence rates of type-1 gNETs per person-year. Due to low heterogeneity (I^2^ = 42.1%), a fixed-effects model was used to calculate and analyze the pooled incidence rate. As shown in Figure 4, the pooled incidence rate of type-1 gNETs in AMAG patients was 0.83% (95% CI: 0.56–1.15) per person-year.

### 3.5. Experience from Our Center and Unusual Gastric Lesions

A previous published study from our center has identified a high coexisting rate of gastric neoplasms with AMAG: 5.9% of AMAG patients was found with early gastric cancer, 3.7% was found with gastric LGD or adenoma, 37% was found with type-1 gNETs, and 31.9% was found with gastric hyperplastic polyps (GHPs) [22]. In addition, part of the GHPs have the potential risk of undergoing neoplastic transformation [23]. Further follow-up of our AMAG cohort is ongoing, and we would like to present three distinctive cases here.

**Figure 5:**
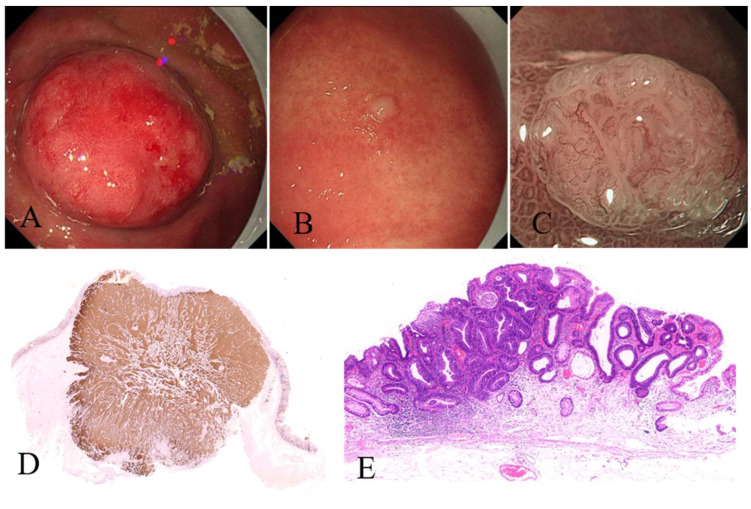
Adenocarcinoma and type-1 gNET arising from the background of AMAG. (**A**) Protruding lesion in the upper corpus, (**B**) white nodule in the lower corpus, (**C**) ME-NBI observation of the nodule showing clear demarcation line with irregular microvessels and microstructure on the surface, (**D**) immunohistochemical stain for Synaptophysin in the protruding lesion (×4), (**E**) HE stain of the white nodule in the lower corpus showing early well-differentiated adenocarcinoma (×4).

**Case 1:** A 48-year-old female patient who presented as anemia and Hashimoto’s disease for 5 years was eventually diagnosed with AMAG according to positive anti-parietal cell antibodies, hypergastrinemia and progressive mucosal atrophy in the corpus and fundus. Helicobacter pylori (*H. pylori*) was tested negative. EGD showed atrophic gastritis in the fundus and corpus. As shown in Figure 5, a 25 mm × 25 mm protruding lesion was detected in the upper corpus (A), and a 6 mm × 5 mm white nodule was detected in the lower corpus(B). Magnifying endoscopy-narrow bind imaging (ME-NBI) showed clear demarcation line with irregular microvessels and microstructure on the surface of the nodule (C). The protruding lesion was removed by laparoscopic local resection, and pathology confirmed it was a type-1 gNET (G2, Ki67 3%) invading the muscularis propria (D, immunohistochemical stain for Synaptophysin, ×4). The white nodule in the lower corpus was removed by endoscopic submucosal dissection (ESD), and pathology indicated it was an early well-differentiated adenocarcinoma (E, HE stain, ×4).

**Figure 6:**
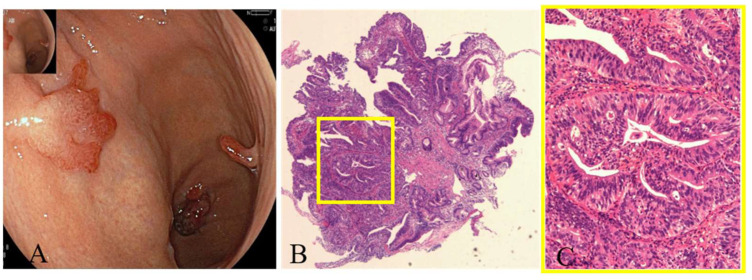
Malignant transformation of a GHP arising from the background of AMAG. (**A**) EGD showing multiple gastric hyperplastic polyps, (**B**,**C**) pathology revealing moderately differentiated adenocarcinoma at the tip of one of the GHPs (yellow square, HE stain; (**B**) ×4; and (**C**) ×20).

**Case 2:** A 70-year-old female patient who presented as anemic for 2 years was diagnosed with AMAG. H. pylori was tested negative. EGD showed severe atrophic gastritis in the fundus and corpus (Figure 6). Six gastric hyperplastic polyps (GHPs) scattered in the fundus, corpus and antrum. After removal by ESD or endoscopic mucosal resection (EMR), pathology revealed moderately differentiated adenocarcinoma at the tip of one of the GHPs.

**Figure 7:**
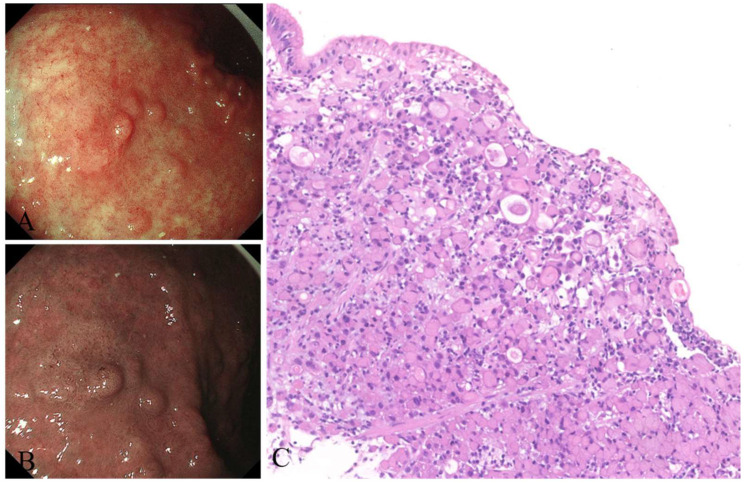
Signet-ring-cell carcinoma arising from AMAG. (**A**) White light and (**B**) NBI showed a depressed lesion in the corpus, and (**C**) pathology revealed signet-ring-cell carcinoma (HE stain, (**C**) ×10).

**Case 3:** A 65-year-old female patient who had Hashimoto’s disease in the past for 5 years was eventually diagnosed with AMAG. *H. pylori* was tested negative. EGD showed severe atrophic gastritis in the fundus and corpus (Figure 7). A 18 mm × 12 mm depressed lesion with discoloration was detected in the middle of the corpus. NBI showed clearly a demarcation line with irregular microvessels on the surface of the lesion. The lesion was removed by ESD and pathology revealed signet-ring-cell carcinoma.

## 4. Discussion

A number of autoimmune conditions have been shown to associate with an increased risk of gastric cancer, including AMAG and PA, as well as frequently co-occuring conditions such as autoimmune thyroiditis, type 1 diabetes mellitus, Addison disease and vitiligo [24]. However, epidemiological evidence for contribution of autoimmunity to gastric carcinogenesis is limited. In order to provide additional evidence for this topic, we conducted this systematic review to describe the incidence rate of gastric lesions associated with AMAG.

Our study showed that the pooled annual incidence of gastric cancer in AMAG patients was 0.14%. Accordingly, the overall gastric cancer relative risk in AMAG was 11.05. These results showed a similar trend compared to a previous published meta-analysis, which demonstrated a gastric cancer incidence rate of 0.27% per person-year in PA patients and relative risk of 6.8 compared with general population [4]. The increased relative risk might be related to the overall decreasing trend in global gastric cancer incidence [25]. Interestingly, cancer registration data have indicated an unexpected increasing incidence in recent generations, along with a reversal of male predominance and a decline in *H. pylori* infection rates, suggesting that gastric cancer related to autoimmunity might be on the rise [26,27,28].

The risk of developing gastric cancer in AMAG patients is not negligible. However, owing to the high rate of asymptomatic disease course, the prevalence of AMAG and gastric cancer associated with AMAG both seems to have been underestimated. A number of studies have reported that a significant proportion of AMAG patients have already developed gastric cancer at the time of diagnosis [22,29,30,31]. Therefore, identifying AMAG and concurrent gastric lesions at early stages of the disease is extremely important. Through rigorous analysis of EGD characteristics, a Japanese study suggested that corpus pan-atrophy, remnant oxyntic mucosa, scattered minute whitish protrusions and sticky adherent dense mucus were important evidences for the diagnosis of AMAG [31]. Interestingly, due to the high incidence of gastric lesions, type-1 gNETs, hyperplastic polyps and even adenocarcinomas were also considered as diagnostic clues for AMAG [31]. Another Japanese study summarized clinicopathological characteristics of 24 early gastric cancer associated with AMAG and concluded that protruded types, larger tumor sizes, upper locations and the papillary pathology type were more likely observed in the AMAG group [32].

With the increased popularity of EGD, more gastric precancerous lesions have been detected. Our study revealed a pooled gastric LGD incidence rate per person-year of 0.52%, which was more than three times of the incidence rate of GC in AMAG patients. Malignant transformation is the major concern for precancerous lesions such as LGD. According to a recent published meta-analysis, the incidence rate of GC was 11.25 per 1000 person-years among patients with LGD lesions [33]. Therefore, these patients should be monitored carefully by long-term surveillance programs.

The severity of mucosal atrophy is associated with the risk of gastric cancer development. The OLGA staging system widely used for scoring severity of atrophy in chronic atrophic gastritis has also been applied to AMAG. Similar to chronic atrophic gastritis, OLGA stages Ⅲ–IV were related to high risk of GC development [34]. According to Rugge and colleagues, after a mean follow-up of 54 months, the OLGA stage was significantly increased in 22% of patients [35]. In contrast, the OLGIM staging system based on intestinal metaplasia was not considered suitable for AMAG staging. Scholars suggest that the OLGIM causes underestimation of grading since it does not consider pseudopyloric metaplasia [34]. Therefore, OLGA staging could provide information for estimation of GC development during AMAG surveillance.

Type-1 gNET is another common gastric lesion related to AMAG. Hypergastrinemia leads to ECL cell hyperplasia, which gradually progresses to gNET development. Our study showed a pooled type-1 gNET incidence rate per person-year of 0.83% in AMAG patients. Similarly, a case-control study based on data extracted from the Surveillance, Epidemiology, and End Results-Medicare database reported an odds ratio of 11.43 for patients with PA to develop type-1 gNET [36]. Chromogranin A (CgA) level greater than 61U/L, male gender and presence of intestinal metaplasia have been identified as significant risk factors for the development of type-1 gNET [37]. Type-1 gNETs generally have a low risk of metastasis and favorable prognosis. Although type-1 gNETs often present as a recurring disease with a median recurrence interval of 24 months after resection, a 100% 5-year survival can be achieved with endoscopic resection [38,39]. Somatostatin analogue therapy has been shown useful to reduce recurrence, which can be monitored by gastrin and CgA levels [40,41]. Moreover, according to Case 1 from our center, the possibility of coexistence with gastric adenocarcinoma needs to be taken into account.

Gastric hyperplastic polyps are found in more than 20% of AMAG cases [42], but GHPs were not the lesion of interest in most studies; therefore, its incidence rate was not calculated in this meta-analysis. GHPs are generally benign, but as presented in Case 2, they have a potential risk of malignant transformation. It has been reported that 1.9–19% of GHPs exhibit dysplasia, with the condition occurring more frequently in GHPs larger than 1 cm and in those with a pedunculated shape [43,44]. According to experience from our center, dysplasia or adenocarcinoma was found within 2.7% of the GHPs, and anemia significantly increased the risk for GHPs to undergo neoplastic transformation [23].

The mechanisms underlying the development of gastric neoplasms in AMAG patients are still unclear, and chronic immune responses appear to play an important role. Previous studies have identified the important roles of cytokines in tumorigenesis in AMAG patients. It is now well accepted that T helper 1 (Th1) cells secreting interferon (IFN)-γ play a pivotal role in AMAG [45]. A recent study using a TCR transgenetic mouse model (TxA23) that generates CD4+ T cells autoreactive against H^+^/K^+^-ATPase mimicking AMAG further clarified the role of IFN-γ in carcinogenesis [46]. Interleukin-17 (IL-17) is also known as a tumorigenesis promoter in gastric cancer. Studies have reported that GC patients exhibit higher levels of IL-17 in both serum and cancer tissues, and advanced Th17 cell infiltration in cancer tissue can be observed in gastric cancer patients [47,48]. Of note, an Italian group recently showed that serum IL-17 subfamily (IL-17A, IL-17F, etc.) levels were significantly elevated in AMAG patients and confirmed that high levels of IL-17A and IL-17F were produced by gastric lamina propria mononuclear cells activated by H^+^/K^+^-ATPase [49].

Apart from inflammation-related pathways, other possible mechanisms of carcinogenesis in AMAG have also been demonstrated. A study focusing on proteomics profiles of AMAG revealed decreased abundance of proteins related to “tricarboxylic acid (TCA) cycle” in the gastric corpus and increased abundance of proteins related to “structural molecule activity” and “cadherin binding involved in cell–cell adhesion”, possibly indicating decreased respiratory capacity in the atrophic background and increased synthesis of intercellular adhesion molecules in attempt to counteract atrophy of the gastric mucosa [50]. The composition of gastric microbiota in AMAG has also become a hot topic in recent years. Parsons et al. observed a greater bacterial abundance and relatively higher microbial diversity in AMAG compared to normal stomachs [51]. Culture testing revealed high prevalence of Klebsiella pneumoniae and alpha-streptococcus in AMAG patients [52], while 16S rRNA sequencing demonstrated significantly higher proportions of Streptococcus, Selenomonas, Granulicatella and Bacillus in AMAG patients [53]. Although the composition of gastric microbiota has not been thoroughly investigated, these findings shed light on possible mechanisms of gastric carcinogenesis in AMAG.

Our study has several limitations. First of all, the asymptomatic nature of AMAG leads to inability to determine the duration of gastric mucosal atrophy. AMAG may have been present in some patients for decades before they were diagnosed. Secondly, the nomenclature and diagnostic criteria of AMAG have evolved over time, and diagnostic criteria vary across studies. Third, most prospective studies have relatively small numbers of subjects and limited duration of follow-up.

## 5. Conclusions

AMAG is related to increased risk of gastric neoplastic changes. Based on this systematic review, patients with AMAG had an incidence rate of 0.14% per person-year and an estimated 11.05-fold RR for GC. Additionally, our study showed a pooled gastric LGD incidence rate of 0.52% per person-year, and pooled gastric type-1 gNETs incidence rate of 0.83% per person-year. The cases shared from our center underscore the potential for malignant transformation of precancerous lesions and reiterate the importance of careful EGD surveillance. The etiology of cancer development in AMAG remains unclear, and further studies are needed.

## Figures and Tables

**Table 1:** Characteristics of Included Studies.

Author	Year	Country	Sources of Selection of Participants	Criteria for Diagnosis of AIG	Number of Patients (n)	Duration of Follow-Up (Years)	Type of Follow-Up	Methods of Follow-Up	Person-years	Age of Patients (Mean or Median)	Female (%)	Methods for Identification of Neoplasms	Cases of Gastric Neoplasms	Incidence of GC (%)
Rugge et al. [9]	2022	Italy	Single-center	Serology, histology	211	7.5	Active	Gastroscopy	1583	55.7	75.8	Histology	GC 0 LGD 5 Type-1 gNET 11	0
Esposito et al. [10]	2021	Italy	Single-center	Histology	122	3	Active	Gastroscopy	366	68	73.0	Histology	GC 3 LGD 2 Type-1 gNET 6	0.82
Miceli et al. [11]	2019	Italy	Single-center	Histology	270	3	Active	Gastroscopy	1164	60.3	70.6	Histology	HGD/GC 3 LGD 4 Type-1 gNET 7	0.26
Mahmud et al. [12]	2019	USA	Single-center	Endoscopic, histology	59	1.89	Not active	Gastroscopy	141	63.5	80.7	Histology	GC 2	1.42
Chan et al. [13]	2008	China	Single-center	PA	199	5.13	Active	Gastroscopy performed in 46/199	1021	73.03	64.3	Histology	GC 4	0.39
Bresky et al. [14]	2003	Spain	Single-center	PA	68	-	Active	Gastroscopy	544	62	57	Histology	GC 0 LGC 3	0
Kokkola et al. [15]	1998	Finland	Single-center	PA	71	12.2	Active	Gastroscopy	869	59	59.2	Histology	GC 2 Type-1 gNET 11	0.23
Borch et al. [16]	1986	Sweden	Single-center	PA	61	2.67	Active	Gastroscopy	163	68	60	Histology	GC 0 LGD 5	0
Schafer et al. [17]	1985	USA	Regional	PA	152	12.5	Non-active	NA	1555	69	63.2	Autopsy	GC 1	0.06
Elsborg et al. [18]	1977	Denmark	Single-center	PA	68	9.5	Active	Gastroscopy	263	65	61.8	Histology	GC 1	0.38
Registration studies														
Ye et al. [19]	2003	Sweden	Swedish Inpatient Register	PA (ICD)	21265	7.1	Not active	Registry data	161672	74.3	60.3	Nationwide Register of Causes of Death	GC 230	0.14
Mellemkjaerl et al. [20]	1997	Denmark	National registration study	PA (ICD)	5072	5.1	Not active	Registry	25768	71-73	66	Danish Cancer Registry	GC 50	0.19
Brinton et al. [21]	1989	USA	Veterans Administration hospitalization records	PA (ICD)	5161	6.8	Not active	Hospitalization records	34915	67.6	0	Hospitalization records	GC 31	0.09

## Data Availability

The authors declare that the data used to conduct the research are available in the main body of the manuscript and in the Supplementary Material attached.

## Supplementary Materials

The following supporting information can be downloaded at: https://www.mdpi.com/article/10.3390/jcm12031062/s1, Table S1. Search strategy; Table S2. Quality assessment of included studies; Table S3. Publication bias assessment; Figure S1. Sensitivity analysis.

## References

[1] Mårdh S., Song Y.H. (1989). Characterization of antigenic structures in auto-immune atrophic gastritis with pernicious anaemia. The parietal cell H,K-ATPase and the chief cell pepsinogen are the two major antigens. Acta Physiol. Scand..

[2] Lenti M.V., Rugge M., Lahner E., Miceli E., Toh B.H., Genta R.M., De Block C., Hershko C., Di Sabatino A. (2020). Autoimmune gastritis. Nat. Rev. Dis. Prim..

[3] Bizzaro N., Antico A. (2014). Diagnosis and classification of pernicious anemia. Autoimmun. Rev..

[4] Vannella L., Lahner E., Osborn J., Annibale B. (2013). Systematic review: Gastric cancer incidence in pernicious anaemia. Aliment. Pharmacol. Ther..

[5] Kaltsas G., Grozinsky-Glasberg S., Alexandraki K.I., Thomas D., Tsolakis A.V., Gross D., Grossman A.B. (2014). Current concepts in the diagnosis and management of type 1 gastric neuroendocrine neoplasms. Clin. Endocrinol..

[6] Page M.J., McKenzie J.E., Bossuyt P.M., Boutron I., Hoffmann T.C., Mulrow C.D., Shamseer L., Tetzlaff J.M., Akl E.A., Brennan S.E. (2021). The PRISMA 2020 statement: An updated guideline for reporting systematic reviews. BMJ.

[7] Munn Z., Barker T.H., Moola S., Tufanaru C., Stern C., McArthur A., Stephenson M., Aromataris E. (2020). Methodological quality of case series studies: An introduction to the JBI critical appraisal tool. JBI Evid. Synth..

[8] Globocan 2020: Estimated Cancer Incidence, Mortality and Prevalence Worldwide in 2020. https://gco.iarc.fr.

[9] Rugge M., Bricca L., Guzzinati S., Sacchi D., Pizzi M., Savarino E., Farinati F., Zorzi M., Fassan M., Dei Tos A.P. (2022). Autoimmune gastritis: Long-term natural history in naive Helicobacter pylori-negative patients. Gut.

[10] Esposito G., Dilaghi E., Cazzato M., Pilozzi E., Conti L., Carabotti M., Di Giulio E., Annibale B., Lahner E. (2021). Endoscopic surveillance at 3 years after diagnosis, according to European guidelines, seems safe in patients with atrophic gastritis in a low-risk region. Dig. Liver Dis. Off. J. Ital. Soc. Gastroenterol. Ital. Assoc. Study Liver.

[11] Miceli E., Vanoli A., Lenti M.V., Klersy C., Di Stefano M., Luinetti O., Caccia Dominioni C., Pisati M., Staiani M., Gentile A. (2019). Natural history of autoimmune atrophic gastritis: A prospective, single centre, long-term experience. Aliment. Pharmacol. Ther..

[12] Mahmud N., Stashek K., Katona B.W., Tondon R., Shroff S.G., Roses R., Furth E.E., Metz D.C. (2019). The incidence of neoplasia in patients with autoimmune metaplastic atrophic gastritis: A renewed call for surveillance. Ann. Gastroenterol..

[13] Chan J.C., Liu H.S., Kho B.C., Lau T.K., Li V.L., Chan F.H., Leong I.S., Pang H.K., Lee C.K., Liang Y.S. (2008). Longitudinal study of Chinese patients with pernicious anaemia. Postgrad. Med. J..

[14] Bresky G., Mata A., Llach J., Ginis M.A., Pellisi M., Soria M.T., Fernandez-Esparrach G., Mondelo F., Bordas J.M. (2003). Endoscopic findings in a biennial follow-up program in patients with pernicious anemia. Hepato-Gastroenterol..

[15] Kokkola A., Sjöblom S.M., Haapiainen R., Sipponen P., Puolakkainen P., Järvinen H. (1998). The risk of gastric carcinoma and carcinoid tumours in patients with pernicious anaemia. A prospective follow-up study. Scand. J. Gastroenterol..

[16] Borch K. (1986). Epidemiologic, clinicopathologic, and economic aspects of gastroscopic screening of patients with pernicious anemia. Scand. J. Gastroenterol..

[17] Schafer L.W., Larson D.E., Melton L.J., Higgins J.A., Zinsmeister A.R. (1985). Risk of development of gastric carcinoma in patients with pernicious anemia: A population-based study in Rochester, Minnesota. Mayo Clin. Proc..

[18] Elsborg L., Andersen D., Myhere-Jensen O., Bastrup-Madsen P. (1977). Gastric mucosal polyps in pernicious anaemia. Scand. J. Gastroenterol..

[19] Ye W., Nyrén O. (2003). Risk of cancers of the oesophagus and stomach by histology or subsite in patients hospitalised for pernicious anaemia. Gut.

[20] Mellemkjaer L., Gridley G., Møller H., Hsing A.W., Linet M.S., Brinton L.A., Olsen J.H. (1996). Pernicious anaemia and cancer risk in Denmark. Br. J. Cancer.

[21] Brinton L.A., Gridley G., Hrubec Z., Hoover R., Fraumeni J.F. (1989). Cancer risk following pernicious anaemia. Br. J. Cancer.

[22] Hu H., Li R., Shao L., Zhang Q., Xu R., Zhang S. (2022). Gastric lesions in patients with autoimmune metaplastic atrophic gastritis: A retrospective study in a single center. Scand. J. Gastroenterol..

[23] Hu H., Zhang Q., Chen G., Pritchard D.M., Zhang S. (2020). Risk factors and clinical correlates of neoplastic transformation in gastric hyperplastic polyps in Chinese patients. Sci. Rep..

[24] Song M., Latorre G., Ivanovic-Zuvic D., Camargo M.C., Rabkin C.S. (2019). Autoimmune Diseases and Gastric Cancer Risk: A Systematic Review and Meta-Analysis. Cancer Res. Treat..

[25] Sung H., Ferlay J., Siegel R.L., Laversanne M., Soerjomataram I., Jemal A., Bray F. (2021). Global Cancer Statistics 2020: GLOBOCAN Estimates of Incidence and Mortality Worldwide for 36 Cancers in 185 Countries. CA Cancer J. Clin..

[26] Luo G., Zhang Y., Guo P., Wang L., Huang Y., Li K. (2017). Global patterns and trends in stomach cancer incidence: Age, period and birth cohort analysis. Int. J. Cancer.

[27] Song M., Rabkin C.S., Camargo M.C. (2018). Gastric Cancer: An Evolving Disease. Curr. Treat. Options Gastroenterol..

[28] Coati I., Fassan M., Farinati F., Graham D.Y., Genta R.M., Rugge M. (2015). Autoimmune gastritis: Pathologist’s viewpoint. World J. Gastroenterol..

[29] Park J.Y., Cornish T.C., Lam-Himlin D., Shi C., Montgomery E. (2010). Gastric lesions in patients with autoimmune metaplastic atrophic gastritis (AMAG) in a tertiary care setting. Am. J. Surg. Pathol..

[30] Zhang H., Jin Z., Cui R., Ding S., Huang Y., Zhou L. (2017). Autoimmune metaplastic atrophic gastritis in chinese: A study of 320 patients at a large tertiary medical center. Scand. J. Gastroenterol..

[31] Terao S., Suzuki S., Yaita H., Kurahara K., Shunto J., Furuta T., Maruyama Y., Ito M., Kamada T., Aoki R. (2020). Multicenter study of autoimmune gastritis in Japan: Clinical and endoscopic characteristics. Dig. Endosc..

[32] Kitamura S., Muguruma N., Okamoto K., Kagemoto K., Kida Y., Mitsui Y., Ueda H., Kawaguchi T., Miyamoto H., Sato Y. (2021). Clinicopathological characteristics of early gastric cancer associated with autoimmune gastritis. JGH Open.

[33] Akbari M., Kardeh B., Tabrizi R., Ahmadizar F., Lankarani K.B. (2019). Incidence Rate of Gastric Cancer Adenocarcinoma in Patients With Gastric Dysplasia: A Systematic Review and Meta-Analysis. J. Clin. Gastroenterol..

[34] Massironi S., Zilli A., Elvevi A., Invernizzi P. (2019). The changing face of chronic autoimmune atrophic gastritis: An updated comprehensive perspective. Autoimmun. Rev..

[35] Rugge M., Fassan M., Pizzi M., Zorzetto V., Maddalo G., Realdon S., De Bernard M., Betterle C., Cappellesso R., Pennelli G. (2012). Autoimmune gastritis: Histology phenotype and OLGA staging. Aliment. Pharmacol. Ther..

[36] Murphy G., Dawsey S.M., Engels E.A., Ricker W., Parsons R., Etemadi A., Lin S.W., Abnet C.C., Freedman N.D. (2015). Cancer Risk After Pernicious Anemia in the US Elderly Population. Clin. Gastroenterol. Hepatol. Off. Clin. Pract. J. Am. Gastroenterol. Assoc..

[37] Campana D., Ravizza D., Ferolla P., Faggiano A., Grimaldi F., Albertelli M., Ricci C., Santini D., Brighi N., Fazio N. (2017). Risk factors of type 1 gastric neuroendocrine neoplasia in patients with chronic atrophic gastritis. A retrospective, multicentre study. Endocrine.

[38] Delle Fave G., O’Toole D., Sundin A., Taal B., Ferolla P., Ramage J.K., Ferone D., Ito T., Weber W., Zheng-Pei Z. (2016). ENETS Consensus Guidelines Update for Gastroduodenal Neuroendocrine Neoplasms. Neuroendocrinology.

[39] Panzuto F., Campana D., Massironi S., Faggiano A., Rinzivillo M., Lamberti G., Sciola V., Lahner E., Manuzzi L., Colao A. (2019). Tumour type and size are prognostic factors in gastric neuroendocrine neoplasia: A multicentre retrospective study. Dig. Liver Dis. Off. J. Ital. Soc. Gastroenterol. Ital. Assoc. Study Liver.

[40] Massironi S., Zilli A., Fanetti I., Ciafardini C., Conte D., Peracchi M. (2015). Intermittent treatment of recurrent type-1 gastric carcinoids with somatostatin analogues in patients with chronic autoimmune atrophic gastritis. Dig. Liver Dis. Off. J. Ital. Soc. Gastroenterol. Ital. Assoc. Study Liver.

[41] Massironi S., Zilli A., Conte D. (2015). Somatostatin analogs for gastric carcinoids: For many, but not all. World J. Gastroenterol..

[42] Kishino M., Nonaka K. (2022). Endoscopic Features of Autoimmune Gastritis: Focus on Typical Images and Early Images. J. Clin. Med..

[43] Waldum H., Fossmark R. (2021). Gastritis, Gastric Polyps and Gastric Cancer. Int. J. Mol. Sci..

[44] Kang H.M., Oh T.H., Seo J.Y., Joen T.J., Seo D.D., Shin W.C., Choi W.C., Kim J.Y. (2011). Clinical factors predicting for neoplastic transformation of gastric hyperplastic polyps. Korean J. Gastroenterol. = Taehan Sohwagi Hakhoe Chi.

[45] D’Elios M.M., Bergman M.P., Azzurri A., Amedei A., Benagiano M., De Pont J.J., Cianchi F., Vandenbroucke-Grauls C.M., Romagnani S., Appelmelk B.J. (2001). H(+),K(+)-atpase (proton pump) is the target autoantigen of Th1-type cytotoxic T cells in autoimmune gastritis. Gastroenterology.

[46] Osaki L.H., Bockerstett K.A., Wong C.F., Ford E.L., Madison B.B., DiPaolo R.J., Mills J.C. (2019). Interferon-gamma directly induces gastric epithelial cell death and is required for progression to metaplasia. J. Pathol..

[47] Meng X.Y., Zhou C.H., Ma J., Jiang C., Ji P. (2012). Expression of interleukin-17 and its clinical significance in gastric cancer patients. Med. Oncol..

[48] Maruyama T., Kono K., Mizukami Y., Kawaguchi Y., Mimura K., Watanabe M., Izawa S., Fujii H. (2010). Distribution of Th17 cells and FoxP3(+) regulatory T cells in tumor-infiltrating lymphocytes, tumor-draining lymph nodes and peripheral blood lymphocytes in patients with gastric cancer. Cancer Sci..

[49] Della Bella C., Antico A., Panozzo M.P., Capitani N., Petrone L., Benagiano M., D’Elios S., Sparano C., Azzurri A., Pratesi S. (2022). Gastric Th17 Cells Specific for H(+)/K(+)-ATPase and Serum IL-17 Signature in Gastric Autoimmunity. Front. Immunol..

[50] Repetto O., De Re V., Giuffrida P., Lenti M.V., Magris R., Venerito M., Steffan A., Di Sabatino A., Cannizzaro R. (2021). Proteomics signature of autoimmune atrophic gastritis: Towards a link with gastric cancer. Gastric. Cancer.

[51] Parsons B.N., Ijaz U.Z., D’Amore R., Burkitt M.D., Eccles R., Lenzi L., Duckworth C.A., Moore A.R., Tiszlavicz L., Varro A. (2017). Comparison of the human gastric microbiota in hypochlorhydric states arising as a result of Helicobacter pylori-induced atrophic gastritis, autoimmune atrophic gastritis and proton pump inhibitor use. PLoS Pathog..

[52] Furuta T., Baba S., Yamade M., Uotani T., Kagami T., Suzuki T., Tani S., Hamaya Y., Iwaizumi M., Osawa S. (2018). High incidence of autoimmune gastritis in patients misdiagnosed with two or more failures of H. pylori eradication. pylori eradication. Aliment. Pharmacol. Ther..

[53] Tsuboi M., Niikura R., Hayakawa Y., Hirata Y., Ushiku T., Koike K. (2020). Distinct Features of Autoimmune Gastritis in Patients with Open-Type Chronic Gastritis in Japan. Biomedicines.

