# Supplementary material for: Incidence of Gastric Neoplasms Arising from Autoimmune Metaplastic Atrophic Gastritis: A Systematic Review and Case Reports
Source: J Clin Med. 2023 Jan 30;12(3):1062. doi: 10.3390/jcm12031062 (PMC9918256; doi:10.3390/jcm12031062)

Table S1. Search strategy

Search terms	**Details**	**Total**
Autoimmune gastritis gastric cancer	("gastritis, atrophic"[MeSH Terms] OR ("gastritis"[All Fields] AND "atrophic"[All Fields]) OR "atrophic gastritis"[All Fields] OR ("autoimmune"[All Fields] AND "gastritis"[All Fields]) OR "autoimmune gastritis"[All Fields]) AND ("stomach neoplasms"[MeSH Terms] OR ("stomach"[All Fields] AND "neoplasms"[All Fields]) OR "stomach neoplasms"[All Fields] OR ("gastric"[All Fields] AND "cancer"[All Fields]) OR "gastric cancer"[All Fields])	3,253
Autoimmune gastritis gastric adenocarcinoma	("gastritis, atrophic"[MeSH Terms] OR ("gastritis"[All Fields] AND "atrophic"[All Fields]) OR "atrophic gastritis"[All Fields] OR ("autoimmune"[All Fields] AND "gastritis"[All Fields]) OR "autoimmune gastritis"[All Fields]) AND ("stomach"[MeSH Terms] OR "stomach"[All Fields] OR "gastric"[All Fields]) AND ("adenocarcinoma"[MeSH Terms] OR "adenocarcinoma"[All Fields])	838
Autoimmune gastritis gastric neoplas*	("gastritis, atrophic"[MeSH Terms] OR ("gastritis"[All Fields] AND "atrophic"[All Fields]) OR "atrophic gastritis"[All Fields] OR ("autoimmune"[All Fields] AND "gastritis"[All Fields]) OR "autoimmune gastritis"[All Fields]) AND (gastric neoplasia[All Fields] OR gastric neoplasias[All Fields] OR gastric neoplasm[All Fields] OR gastric neoplasms[All Fields])	2583
Autoimmune gastritis gastric carcinoma	("gastritis, atrophic"[MeSH Terms] OR ("gastritis"[All Fields] AND "atrophic"[All Fields]) OR "atrophic gastritis"[All Fields] OR ("autoimmune"[All Fields] AND "gastritis"[All Fields]) OR "autoimmune gastritis"[All Fields]) AND ("stomach"[MeSH Terms] OR "stomach"[All Fields] OR "gastric"[All Fields]) AND ("carcinoma"[MeSH Terms] OR "carcinoma"[All Fields])	1110
Autoimmune gastritis gastric tumor	("gastritis, atrophic"[MeSH Terms] OR ("gastritis"[All Fields] AND "atrophic"[All Fields]) OR "atrophic gastritis"[All Fields] OR ("autoimmune"[All Fields] AND "gastritis"[All Fields]) OR "autoimmune gastritis"[All Fields]) AND ("stomach neoplasms"[MeSH Terms] OR ("stomach"[All Fields] AND "neoplasms"[All Fields]) OR "stomach neoplasms"[All Fields] OR ("gastric"[All Fields] AND "tumor"[All Fields]) OR "gastric tumor"[All Fields])	2637
Autoimmune gastritis gastric NET	("gastritis, atrophic"[MeSH Terms] OR ("gastritis"[All Fields] AND "atrophic"[All Fields]) OR "atrophic gastritis"[All Fields] OR ("autoimmune"[All Fields] AND "gastritis"[All Fields]) OR "autoimmune gastritis"[All Fields]) AND ("stomach"[MeSH Terms] OR "stomach"[All Fields] OR "gastric"[All Fields]) AND ("neuroendocrine tumors"[MeSH Terms] OR "carcinoid tumor"[MeSH Terms] OR "neuroendocrine"[All Fields])	413

Autoimmune gastritis dysplasia	("gastritis, atrophic"[MeSH Terms] OR ("gastritis"[All Fields] AND "atrophic"[All Fields]) OR "atrophic gastritis"[All Fields] OR ("autoimmune"[All Fields] AND "gastritis"[All Fields]) OR "autoimmune gastritis"[All Fields]) AND ("stomach"[MeSH Terms] OR "stomach"[All Fields] OR "gastric"[All Fields]) AND “dysplasia”[All Fields]	682
Autoimmune gastritis Hyperplastic polyp	("gastritis, atrophic"[MeSH Terms] OR ("gastritis"[All Fields] AND "atrophic"[All Fields]) OR "atrophic gastritis"[All Fields] OR ("autoimmune"[All Fields] AND "gastritis"[All Fields]) OR "autoimmune gastritis"[All Fields]) AND ("stomach"[MeSH Terms] OR "stomach"[All Fields] OR "gastric"[All Fields]) AND ("polyps"[MeSH Terms] OR "polyps"[All Fields] OR "polyposis"[All Fields])	272
Atrophic gastritis gastric cancer	("gastritis, atrophic"[MeSH Terms] OR ("gastritis"[All Fields] AND "atrophic"[All Fields]) OR "atrophic gastritis"[All Fields] OR ("atrophic"[All Fields] AND "gastritis"[All Fields])) AND ("stomach neoplasms"[MeSH Terms] OR ("stomach"[All Fields] AND "neoplasms"[All Fields]) OR "stomach neoplasms"[All Fields] OR ("gastric"[All Fields] AND "cancer"[All Fields]) OR "gastric cancer"[All Fields])	3084
Atrophic gastritis gastric adenocarcinoma	("gastritis, atrophic"[MeSH Terms] OR ("gastritis"[All Fields] AND "atrophic"[All Fields]) OR "atrophic gastritis"[All Fields] OR ("atrophic"[All Fields] AND "gastritis"[All Fields])) AND ("stomach"[MeSH Terms] OR "stomach"[All Fields] OR "gastric"[All Fields]) AND ("adenocarcinoma"[MeSH Terms] OR "adenocarcinoma"[All Fields])	784
Atrophic gastritis gastric neoplas*	("gastritis, atrophic"[MeSH Terms] OR ("gastritis"[All Fields] AND "atrophic"[All Fields]) OR "atrophic gastritis"[All Fields] OR ("atrophic"[All Fields] AND "gastritis"[All Fields])) AND (gastric neoplasia[All Fields] OR gastric neoplasias[All Fields] OR gastric neoplasm[All Fields] OR gastric neoplasms[All Fields])	2473
Atrophic gastritis gastric carcinoma	("gastritis, atrophic"[MeSH Terms] OR ("gastritis"[All Fields] AND "atrophic"[All Fields]) OR "atrophic gastritis"[All Fields] OR ("atrophic"[All Fields] AND "gastritis"[All Fields])) AND ("stomach"[MeSH Terms] OR "stomach"[All Fields] OR "gastric"[All Fields]) AND ("carcinoma"[MeSH Terms] OR "carcinoma"[All Fields])	1063
Atrophic gastritis gastric tumor	("gastritis, atrophic"[MeSH Terms] OR ("gastritis"[All Fields] AND "atrophic"[All Fields]) OR "atrophic gastritis"[All Fields] OR ("atrophic"[All Fields] AND "gastritis"[All Fields])) AND ("stomach neoplasms"[MeSH Terms] OR ("stomach"[All Fields] AND "neoplasms"[All Fields]) OR "stomach neoplasms"[All Fields] OR ("gastric"[All Fields] AND "tumor"[All Fields]) OR "gastric tumor"[All Fields])	2517
Atrophic gastritis gastric NET	("gastritis, atrophic"[MeSH Terms] OR ("gastritis"[All Fields] AND "atrophic"[All Fields]) OR "atrophic gastritis"[All Fields] OR ("atrophic"[All Fields] AND "gastritis"[All Fields])) AND ("stomach"[MeSH Terms] OR "stomach"[All	420

	Fields] OR "gastric"[All Fields]) AND (“neuroendocrine tumors"[MeSH Terms] OR “carcinoid tumor"[MeSH Terms] OR "neuroendocrine"[All Fields] OR “carcinoid”[All Fields])	
Atrophic gastritis dysplasia	("gastritis, atrophic"[MeSH Terms] OR ("gastritis"[All Fields] AND "atrophic"[All Fields]) OR "atrophic gastritis"[All Fields] OR ("atrophic"[All Fields] AND "gastritis"[All Fields])) AND ("stomach"[MeSH Terms] OR "stomach"[All Fields] OR "gastric"[All Fields]) AND “dysplasia”[All Fields]	661
Atrophic gastritis polyp	("gastritis, atrophic"[MeSH Terms] OR ("gastritis"[All Fields] AND "atrophic"[All Fields]) OR "atrophic gastritis"[All Fields] OR ("atrophic"[All Fields] AND "gastritis"[All Fields])) AND ("stomach"[MeSH Terms] OR "stomach"[All Fields] OR "gastric"[All Fields]) AND ("polyps"[MeSH Terms] OR "polyps"[All Fields] OR "polyposis"[All Fields])	247
Type-A gastritis gastric cancer	type-A[All Fields] AND ("gastritis"[MeSH Terms] OR "gastritis"[All Fields]) AND ("stomach neoplasms"[MeSH Terms] OR ("stomach"[All Fields] AND "neoplasms"[All Fields]) OR "stomach neoplasms"[All Fields] OR ("gastric"[All Fields] AND "cancer"[All Fields]) OR "gastric cancer"[All Fields])	120
Type-A gastritis gastric adenocarcinoma	type-A[All Fields] AND ("gastritis"[MeSH Terms] OR "gastritis"[All Fields]) AND ("stomach"[MeSH Terms] OR "stomach"[All Fields] OR "gastric"[All Fields]) AND ("adenocarcinoma"[MeSH Terms] OR "adenocarcinoma"[All Fields])	79
Type-A gastritis gastric neoplas*	type-A[All Fields] AND ("gastritis"[MeSH Terms] OR "gastritis"[All Fields]) AND (gastric neoplasia[All Fields] OR gastric neoplasias[All Fields] OR gastric neoplasm[All Fields] OR gastric neoplasms[All Fields])	111
Type-A gastritis gastric carcinoma	type-A[All Fields] AND ("gastritis"[MeSH Terms] OR "gastritis"[All Fields]) AND ("stomach"[MeSH Terms] OR "stomach"[All Fields] OR "gastric"[All Fields]) AND ("carcinoma"[MeSH Terms] OR "carcinoma"[All Fields])	87
Type-A gastritis gastric tumor	type-A[All Fields] AND ("gastritis"[MeSH Terms] OR "gastritis"[All Fields]) AND ("stomach neoplasms"[MeSH Terms] OR ("stomach"[All Fields] AND "neoplasms"[All Fields]) OR "stomach neoplasms"[All Fields] OR ("gastric"[All Fields] AND "tumor"[All Fields]) OR "gastric tumor"[All Fields])	115
Type-A gastritis gastric NET	type-A[All Fields] AND ("gastritis"[MeSH Terms] OR "gastritis"[All Fields]) AND ("stomach"[MeSH Terms] OR "stomach"[All Fields] OR "gastric"[All Fields]) AND (“neuroendocrine tumors"[MeSH Terms] OR “carcinoid tumor"[MeSH Terms] OR "neuroendocrine"[All Fields] OR “carcinoid”[All Fields])	77

Type-A gastritis dysplasia	type-A[All Fields] AND ("gastritis"[MeSH Terms] OR "gastritis"[All Fields]) AND ("stomach"[MeSH Terms] OR "stomach"[All Fields] OR "gastric"[All Fields]) AND "dysplasia"[All Fields]	14
Type-A gastritis polyp	type-A[All Fields] AND ("gastritis"[MeSH Terms] OR "gastritis"[All Fields]) AND ("stomach"[MeSH Terms] OR "stomach"[All Fields] OR "gastric"[All Fields]) AND ("polyps"[MeSH Terms] OR "polyps"[All Fields] OR "polyposis"[All Fields])	20
Pernicious anemia gastric cancer	("pernicious anaemia"[All Fields] OR "anemia, pernicious"[MeSH Terms] OR ("anemia"[All Fields] AND "pernicious"[All Fields]) OR "pernicious anemia"[All Fields] OR ("pernicious"[All Fields] AND "anemia"[All Fields])) AND ("stomach neoplasms"[MeSH Terms] OR ("stomach"[All Fields] AND "neoplasms"[All Fields]) OR "stomach neoplasms"[All Fields] OR ("gastric"[All Fields] AND "cancer"[All Fields]) OR "gastric cancer"[All Fields])	618
Pernicious anemia gastric adenocarcinoma	("pernicious anaemia"[All Fields] OR "anemia, pernicious"[MeSH Terms] OR ("anemia"[All Fields] AND "pernicious"[All Fields]) OR "pernicious anemia"[All Fields] OR ("pernicious"[All Fields] AND "anemia"[All Fields])) AND ("stomach"[MeSH Terms] OR "stomach"[All Fields] OR "gastric"[All Fields]) AND ("adenocarcinoma"[MeSH Terms] OR "adenocarcinoma"[All Fields])	210
Pernicious anemia gastric neoplas*	("pernicious anaemia"[All Fields] OR "anemia, pernicious"[MeSH Terms] OR ("anemia"[All Fields] AND "pernicious"[All Fields]) OR "pernicious anemia"[All Fields] OR ("pernicious"[All Fields] AND "anemia"[All Fields])) AND (gastric neoplasia[All Fields] OR gastric neoplasias[All Fields] OR gastric neoplasm[All Fields] OR gastric neoplasms[All Fields])	594
Pernicious anemia gastric carcinoma	("pernicious anaemia"[All Fields] OR "anemia, pernicious"[MeSH Terms] OR ("anemia"[All Fields] AND "pernicious"[All Fields]) OR "pernicious anemia"[All Fields] OR ("pernicious"[All Fields] AND "anemia"[All Fields])) AND ("stomach"[MeSH Terms] OR "stomach"[All Fields] OR "gastric"[All Fields]) AND ("carcinoma"[MeSH Terms] OR "carcinoma"[All Fields])	310
Pernicious anemia gastric tumor	("pernicious anaemia"[All Fields] OR "anemia, pernicious"[MeSH Terms] OR ("anemia"[All Fields] AND "pernicious"[All Fields]) OR "pernicious anemia"[All Fields] OR ("pernicious"[All Fields] AND "anemia"[All Fields])) AND ("stomach neoplasms"[MeSH Terms] OR ("stomach"[All Fields] AND "neoplasms"[All Fields]) OR "stomach neoplasms"[All Fields] OR ("gastric"[All Fields] AND "tumor"[All Fields]) OR "gastric tumor"[All Fields])	591

Pernicious anemia gastric NET	("pernicious anaemia"[All Fields] OR "anemia, pernicious"[MeSH Terms] OR ("anemia"[All Fields] AND "pernicious"[All Fields]) OR "pernicious anemia"[All Fields] OR ("pernicious"[All Fields] AND "anemia"[All Fields])) AND ("stomach"[MeSH Terms] OR "stomach"[All Fields] OR "gastric"[All Fields]) AND ("neuroendocrine tumors"[MeSH Terms] OR "carcinoid tumor"[MeSH Terms] OR "neuroendocrine"[All Fields] OR "carcinoid"[All Fields])	197
Pernicious anemia dysplasia	("pernicious anaemia"[All Fields] OR "anemia, pernicious"[MeSH Terms] OR ("anemia"[All Fields] AND "pernicious"[All Fields]) OR "pernicious anemia"[All Fields] OR ("pernicious"[All Fields] AND "anemia"[All Fields])) AND ("stomach"[MeSH Terms] OR "stomach"[All Fields] OR "gastric"[All Fields]) AND "dysplasia"[All Fields]	41
Pernicious anemia polyp	("pernicious anaemia"[All Fields] OR "anemia, pernicious"[MeSH Terms] OR ("anemia"[All Fields] AND "pernicious"[All Fields]) OR "pernicious anemia"[All Fields] OR ("pernicious"[All Fields] AND "anemia"[All Fields])) AND ("stomach"[MeSH Terms] OR "stomach"[All Fields] OR "gastric"[All Fields]) AND ("polyps"[MeSH Terms] OR "polyps"[All Fields] OR "polyposis"[All Fields])	99
Macrocytic anemia gastric cancer	"macrocytic anaemia"[All Fields] OR "anemia, macrocytic"[MeSH Terms] OR ("anemia"[All Fields] AND "macrocytic"[All Fields]) OR "macrocytic anemia"[All Fields] OR ("macrocytic"[All Fields] AND "anemia"[All Fields])) AND ("stomach neoplasms"[MeSH Terms] OR ("stomach"[All Fields] AND "neoplasms"[All Fields]) OR "stomach neoplasms"[All Fields] OR ("gastric"[All Fields] AND "cancer"[All Fields]) OR "gastric cancer"[All Fields])	519
Macrocytic anemia gastric adenocarcinoma	"macrocytic anaemia"[All Fields] OR "anemia, macrocytic"[MeSH Terms] OR ("anemia"[All Fields] AND "macrocytic"[All Fields]) OR "macrocytic anemia"[All Fields] OR ("macrocytic"[All Fields] AND "anemia"[All Fields])) AND ("stomach"[MeSH Terms] OR "stomach"[All Fields] OR "gastric"[All Fields]) AND ("adenocarcinoma"[MeSH Terms] OR "adenocarcinoma"[All Fields])	139
Macrocytic anemia gastric neoplas*	"macrocytic anaemia"[All Fields] OR "anemia, macrocytic"[MeSH Terms] OR ("anemia"[All Fields] AND "macrocytic"[All Fields]) OR "macrocytic anemia"[All Fields] OR ("macrocytic"[All Fields] AND "anemia"[All Fields])) AND (gastric neoplasia[All Fields] OR gastric neoplasias[All Fields] OR gastric neoplasm[All Fields] OR gastric neoplasms[All Fields])	514
Macrocytic anemia gastric carcinoma	"macrocytic anaemia"[All Fields] OR "anemia, macrocytic"[MeSH Terms] OR ("anemia"[All Fields] AND "macrocytic"[All Fields]) OR "macrocytic anemia"[All Fields] OR ("macrocytic"[All Fields] AND "anemia"[All	233

	Fields])) AND ("stomach"[MeSH Terms] OR "stomach"[All Fields] OR "gastric"[All Fields]) AND ("carcinoma"[MeSH Terms] OR "carcinoma"[All Fields])	
Macrocytic anemia gastric tumor	"macrocytic anaemia"[All Fields] OR "anemia, macrocytic"[MeSH Terms] OR ("anemia"[All Fields] AND "macrocytic"[All Fields]) OR "macrocytic anemia"[All Fields] OR ("macrocytic"[All Fields] AND "anemia"[All Fields])) AND ("stomach neoplasms"[MeSH Terms] OR ("stomach"[All Fields] AND "neoplasms"[All Fields]) OR "stomach neoplasms"[All Fields] OR ("gastric"[All Fields] AND "tumor"[All Fields]) OR "gastric tumor"[All Fields])	499
Macrocytic anemia gastric NET	"macrocytic anaemia"[All Fields] OR "anemia, macrocytic"[MeSH Terms] OR ("anemia"[All Fields] AND "macrocytic"[All Fields]) OR "macrocytic anemia"[All Fields] OR ("macrocytic"[All Fields] AND "anemia"[All Fields])) AND ("stomach"[MeSH Terms] OR "stomach"[All Fields] OR "gastric"[All Fields]) AND ("neuroendocrine tumors"[MeSH Terms] OR "carcinoid tumor"[MeSH Terms] OR "neuroendocrine"[All Fields] OR "carcinoid"[All Fields])	111
Macrocytic anemia dysplasia	"macrocytic anaemia"[All Fields] OR "anemia, macrocytic"[MeSH Terms] OR ("anemia"[All Fields] AND "macrocytic"[All Fields]) OR "macrocytic anemia"[All Fields] OR ("macrocytic"[All Fields] AND "anemia"[All Fields])) AND ("stomach"[MeSH Terms] OR "stomach"[All Fields] OR "gastric"[All Fields]) AND "dysplasia"[All Fields]	11
Macrocytic anemia polyp	"macrocytic anaemia"[All Fields] OR "anemia, macrocytic"[MeSH Terms] OR ("anemia"[All Fields] AND "macrocytic"[All Fields]) OR "macrocytic anemia"[All Fields] OR ("macrocytic"[All Fields] AND "anemia"[All Fields])) AND ("stomach"[MeSH Terms] OR "stomach"[All Fields] OR "gastric"[All Fields]) AND ("polyps"[MeSH Terms] OR "polyps"[All Fields] OR "polyposis"[All Fields])	78
cobalamin deficiency gastric cancer	("vitamin b 12 deficiency"[MeSH Terms] OR "vitamin b 12 deficiency"[All Fields] OR ("cobalamin"[All Fields] AND "deficiency"[All Fields]) OR "cobalamin deficiency"[All Fields]) AND ("stomach neoplasms"[MeSH Terms] OR ("stomach"[All Fields] AND "neoplasms"[All Fields]) OR "stomach neoplasms"[All Fields] OR ("gastric"[All Fields] AND "cancer"[All Fields]) OR "gastric cancer"[All Fields])	552
cobalamin deficiency gastric adenocarcinoma	("vitamin b 12 deficiency"[MeSH Terms] OR "vitamin b 12 deficiency"[All Fields] OR ("cobalamin"[All Fields] AND "deficiency"[All Fields]) OR "cobalamin deficiency"[All Fields]) AND ("stomach"[MeSH Terms] OR "stomach"[All Fields] OR "gastric"[All Fields]) AND ("adenocarcinoma"[MeSH Terms] OR "adenocarcinoma"[All Fields])	145

cobalamin deficiency gastric neoplas*	("vitamin b 12 deficiency"[MeSH Terms] OR "vitamin b 12 deficiency"[All Fields] OR ("cobalamin"[All Fields] AND "deficiency"[All Fields]) OR "cobalamin deficiency"[All Fields]) AND (gastric neoplasia[All Fields] OR gastric neoplasias[All Fields] OR gastric neoplasm[All Fields] OR gastric neoplasms[All Fields])	537
cobalamin deficiency gastric carcinoma	("vitamin b 12 deficiency"[MeSH Terms] OR "vitamin b 12 deficiency"[All Fields] OR ("cobalamin"[All Fields] AND "deficiency"[All Fields]) OR "cobalamin deficiency"[All Fields]) AND ("stomach"[MeSH Terms] OR "stomach"[All Fields] OR "gastric"[All Fields]) AND ("carcinoma"[MeSH Terms] OR "carcinoma"[All Fields])	239
cobalamin deficiency gastric tumor	("vitamin b 12 deficiency"[MeSH Terms] OR "vitamin b 12 deficiency"[All Fields] OR ("cobalamin"[All Fields] AND "deficiency"[All Fields]) OR "cobalamin deficiency"[All Fields]) AND ("stomach neoplasms"[MeSH Terms] OR ("stomach"[All Fields] AND "neoplasms"[All Fields]) OR "stomach neoplasms"[All Fields] OR ("gastric"[All Fields] AND "tumor"[All Fields]) OR "gastric tumor"[All Fields])	521
cobalamin deficiency gastric NET	("vitamin b 12 deficiency"[MeSH Terms] OR "vitamin b 12 deficiency"[All Fields] OR ("cobalamin"[All Fields] AND "deficiency"[All Fields]) OR "cobalamin deficiency"[All Fields]) AND ("stomach"[MeSH Terms] OR "stomach"[All Fields] OR "gastric"[All Fields]) AND (“neuroendocrine tumors"[MeSH Terms] OR “carcinoid tumor"[MeSH Terms] OR "neuroendocrine"[All Fields] OR “carcinoid”[All Fields])	118
cobalamin deficiency dysplasia	("vitamin b 12 deficiency"[MeSH Terms] OR "vitamin b 12 deficiency"[All Fields] OR ("cobalamin"[All Fields] AND "deficiency"[All Fields]) OR "cobalamin deficiency"[All Fields]) AND ("stomach"[MeSH Terms] OR "stomach"[All Fields] OR "gastric"[All Fields]) AND “dysplasia”[All Fields]	12
cobalamin deficiency polyp	("vitamin b 12 deficiency"[MeSH Terms] OR "vitamin b 12 deficiency"[All Fields] OR ("cobalamin"[All Fields] AND "deficiency"[All Fields]) OR "cobalamin deficiency"[All Fields]) AND ("stomach"[MeSH Terms] OR "stomach"[All Fields] OR "gastric"[All Fields]) AND ("polyps"[MeSH Terms] OR "polyps"[All Fields] OR "polyposis"[All Fields])	81
Vitamin B12 deficiency gastric cancer	("vitamin b 12 deficiency"[MeSH Terms] OR "vitamin b 12 deficiency"[All Fields]) AND ("stomach neoplasms"[MeSH Terms] OR ("stomach"[All Fields] AND "neoplasms"[All Fields]) OR "stomach neoplasms"[All Fields] OR ("gastric"[All Fields] AND "cancer"[All Fields]) OR "gastric cancer"[All Fields])	547

Vitamin B12 deficiency gastric adenocarcinoma	("vitamin b 12 deficiency"[MeSH Terms] OR "vitamin b 12 deficiency"[All Fields]) AND ("stomach"[MeSH Terms] OR "stomach"[All Fields] OR "gastric"[All Fields]) AND ("adenocarcinoma"[MeSH Terms] OR "adenocarcinoma"[All Fields])	145
Vitamin B12 deficiency gastric neoplas*	("vitamin b 12 deficiency"[MeSH Terms] OR "vitamin b 12 deficiency"[All Fields]) AND (gastric neoplasia[All Fields] OR gastric neoplasias[All Fields] OR gastric neoplasm[All Fields] OR gastric neoplasms[All Fields])	535
Vitamin B12 deficiency gastric carcinoma	("vitamin b 12 deficiency"[MeSH Terms] OR "vitamin b 12 deficiency"[All Fields]) AND ("stomach"[MeSH Terms] OR "stomach"[All Fields] OR "gastric"[All Fields]) AND ("carcinoma"[MeSH Terms] OR "carcinoma"[All Fields])	239
Vitamin B12 deficiency gastric tumor	("vitamin b 12 deficiency"[MeSH Terms] OR "vitamin b 12 deficiency"[All Fields]) AND ("stomach neoplasms"[MeSH Terms] OR ("stomach"[All Fields] AND "neoplasms"[All Fields]) OR "stomach neoplasms"[All Fields] OR ("gastric"[All Fields] AND "tumor"[All Fields]) OR "gastric tumor"[All Fields])	519
Vitamin B12 deficiency gastric NET	("vitamin b 12 deficiency"[MeSH Terms] OR "vitamin b 12 deficiency"[All Fields]) AND ("stomach"[MeSH Terms] OR "stomach"[All Fields] OR "gastric"[All Fields]) AND (“neuroendocrine tumors"[MeSH Terms] OR “carcinoid tumor"[MeSH Terms] OR "neuroendocrine"[All Fields] OR “carcinoid”[All Fields])	118
Vitamin B12 deficiency dysplasia	("vitamin b 12 deficiency"[MeSH Terms] OR "vitamin b 12 deficiency"[All Fields]) AND ("stomach"[MeSH Terms] OR "stomach"[All Fields] OR "gastric"[All Fields]) AND “dysplasia”[All Fields]	12
Vitamin B12 deficiency polyp	("vitamin b 12 deficiency"[MeSH Terms] OR "vitamin b 12 deficiency"[All Fields]) AND ("stomach"[MeSH Terms] OR "stomach"[All Fields] OR "gastric"[All Fields]) AND ("polyps"[MeSH Terms] OR "polyps"[All Fields] OR "polyposis"[All Fields])	81

Intrinsic factor deficiency gastric cancer	("intrinsic factor"[MeSH Terms] OR ("intrinsic"[All Fields] AND "factor"[All Fields]) OR "intrinsic factor"[All Fields]) AND ("deficiency"[Subheading] OR "deficiency"[All Fields]) AND ("stomach neoplasms"[MeSH Terms] OR ("stomach"[All Fields] AND "neoplasms"[All Fields]) OR "stomach neoplasms"[All Fields] OR ("gastric"[All Fields] AND "cancer"[All Fields]) OR "gastric cancer"[All Fields])	34
Intrinsic factor deficiency gastric adenocarcinoma	("intrinsic factor"[MeSH Terms] OR ("intrinsic"[All Fields] AND "factor"[All Fields]) OR "intrinsic factor"[All Fields]) AND ("deficiency"[Subheading] OR "deficiency"[All Fields]) AND ("stomach"[MeSH Terms] OR "stomach"[All Fields] OR "gastric"[All Fields]) AND ("adenocarcinoma"[MeSH Terms] OR "adenocarcinoma"[All Fields])	10
Intrinsic factor deficiency gastric neoplas*	("intrinsic factor"[MeSH Terms] OR ("intrinsic"[All Fields] AND "factor"[All Fields]) OR "intrinsic factor"[All Fields]) AND ("deficiency"[Subheading] OR "deficiency"[All Fields]) AND (gastric neoplasia[All Fields] OR gastric neoplasias[All Fields] OR gastric neoplasm[All Fields] OR gastric neoplasms[All Fields])	26
Intrinsic factor deficiency gastric carcinoma	("intrinsic factor"[MeSH Terms] OR ("intrinsic"[All Fields] AND "factor"[All Fields]) OR "intrinsic factor"[All Fields]) AND ("deficiency"[Subheading] OR "deficiency"[All Fields]) AND ("stomach"[MeSH Terms] OR "stomach"[All Fields] OR "gastric"[All Fields]) AND ("carcinoma"[MeSH Terms] OR "carcinoma"[All Fields])	9
Intrinsic factor deficiency gastric tumor	("intrinsic factor"[MeSH Terms] OR ("intrinsic"[All Fields] AND "factor"[All Fields]) OR "intrinsic factor"[All Fields]) AND ("deficiency"[Subheading] OR "deficiency"[All Fields]) AND ("stomach neoplasms"[MeSH Terms] OR ("stomach"[All Fields] AND "neoplasms"[All Fields]) OR "stomach neoplasms"[All Fields] OR ("gastric"[All Fields] AND "tumor"[All Fields]) OR "gastric tumor"[All Fields])	25
Intrinsic factor deficiency gastric NET	("intrinsic factor"[MeSH Terms] OR ("intrinsic"[All Fields] AND "factor"[All Fields]) OR "intrinsic factor"[All Fields]) AND ("deficiency"[Subheading] OR "deficiency"[All Fields]) AND ("stomach"[MeSH Terms] OR "stomach"[All Fields] OR "gastric"[All Fields]) AND (“neuroendocrine tumors"[MeSH Terms] OR “carcinoid tumor"[MeSH Terms] OR "neuroendocrine"[All Fields] OR “carcinoid”[All Fields])	8

Intrinsic factor deficiency dysplasia	("intrinsic factor"[MeSH Terms] OR ("intrinsic"[All Fields] AND "factor"[All Fields]) OR "intrinsic factor"[All Fields]) AND ("deficiency"[Subheading] OR "deficiency"[All Fields]) AND ("stomach"[MeSH Terms] OR "stomach"[All Fields] OR "gastric"[All Fields]) AND “dysplasia”[All Fields]	1
Intrinsic factor deficiency polyp	("intrinsic factor"[MeSH Terms] OR ("intrinsic"[All Fields] AND "factor"[All Fields]) OR "intrinsic factor"[All Fields]) AND ("deficiency"[Subheading] OR "deficiency"[All Fields]) AND ("stomach"[MeSH Terms] OR "stomach"[All Fields] OR "gastric"[All Fields]) AND ("polyps"[MeSH Terms] OR "polyps"[All Fields] OR "polyposis"[All Fields])	3
'intrinsic factor deficiency':ti,ab,kw AND gastric:ti,ab,kw AND (tumor:ti,ab,kw OR cancer:ti,ab,kw OR neoplasm:ti,ab,kw OR adenocarcinoma:ti,ab,kw OR polyp:ti,ab,kw OR dysplasia:ti,ab,kw OR neuroendocrine:ti,ab,kw)		7
'vitamin b12 deficiency':ti,ab,kw AND gastric:ti,ab,kw AND (tumor:ti,ab,kw OR cancer:ti,ab,kw OR neoplasm:ti,ab,kw OR adenocarcinoma:ti,ab,kw OR polyp:ti,ab,kw OR dysplasia:ti,ab,kw OR neuroendocrine:ti,ab,kw)		91
'cobalamin deficiency':ti,ab,kw AND gastric:ti,ab,kw AND (tumor:ti,ab,kw OR cancer:ti,ab,kw OR neoplasm:ti,ab,kw OR adenocarcinoma:ti,ab,kw OR polyp:ti,ab,kw OR dysplasia:ti,ab,kw OR neuroendocrine:ti,ab,kw)		21
'macrocytic anemia':ti,ab,kw AND gastric:ti,ab,kw AND (tumor:ti,ab,kw OR cancer:ti,ab,kw OR neoplasm:ti,ab,kw OR adenocarcinoma:ti,ab,kw OR polyp:ti,ab,kw OR dysplasia:ti,ab,kw OR neuroendocrine:ti,ab,kw)		31
'autoimmune gastritis':ti,ab,kw AND gastric:ti,ab,kw AND (tumor:ti,ab,kw OR cancer:ti,ab,kw OR neoplasm:ti,ab,kw OR adenocarcinoma:ti,ab,kw OR polyp:ti,ab,kw OR dysplasia:ti,ab,kw OR neuroendocrine:ti,ab,kw)		319
'type-a gastritis':ti,ab,kw AND gastric:ti,ab,kw AND (tumor:ti,ab,kw OR cancer:ti,ab,kw OR neoplasm:ti,ab,kw OR adenocarcinoma:ti,ab,kw OR polyp:ti,ab,kw OR dysplasia:ti,ab,kw OR neuroendocrine:ti,ab,kw)		51
'pernicious anemia':ti,ab,kw AND gastric:ti,ab,kw AND (tumor:ti,ab,kw OR cancer:ti,ab,kw OR neoplasm:ti,ab,kw OR adenocarcinoma:ti,ab,kw OR polyp:ti,ab,kw OR dysplasia:ti,ab,kw OR neuroendocrine:ti,ab,kw)		356

Table S2. Quality assessment of included studies

First Author	Q1	Q2	Q3	Q4	Q5	Q6	Q7	Q8	Q9	Q10	Total QA score
Rugge et al. [9]	YES	YES	YES	YES	NO	YES	YES	YES	YES	YES	9
Esposito et al [10]	YES	YES	YES	YES	YES	YES	YES	YES	YES	YES	10
Miceli et al. [11]	YES	YES	YES	YES	YES	YES	YES	YES	YES	YES	10
Mahmud et al. [12]	YES	YES	YES	YES	YES	YES	YES	YES	YES	YES	10
Chan et al. [13]	YES	YES	YES	YES	YES	YES	YES	YES	YES	YES	10
Ye et al. [19]	YES	YES	YES	YES	YES	YES	NO	YES	YES	YES	9
Bresky et al. [14]	YES	YES	YES	YES	YES	NO	NO	YES	NO	YES	7
Kokkola et al. [15]	YES	YES	YES	YES	NO	YES	NO	YES	YES	YES	8
Mellemkjaerl et al. [20]	YES	YES	YES	YES	YES	YES	NO	NO	YES	YES	8
Brinton et al. [21]	YES	YES	YES	YES	YES	YES	NO	NO	YES	YES	8
Borch et al. [16]	YES	YES	YES	YES	YES	NO	NO	YES	NO	YES	7
Schafer et al. [17]	YES	YES	YES	YES	YES	YES	NO	YES	YES	YES	9
Elsborg et al. [18]	NO	NO	NO	YES	YES	YES	NO	YES	YES	YES	6

Table S3. Publication bias assessment using the funnel plot and The Egger test

	Funnel plot	P value for the Egger test
GC	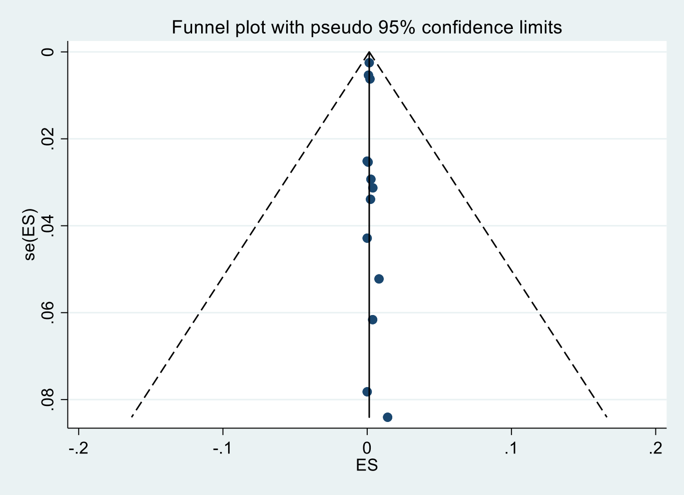	0.183
LGD	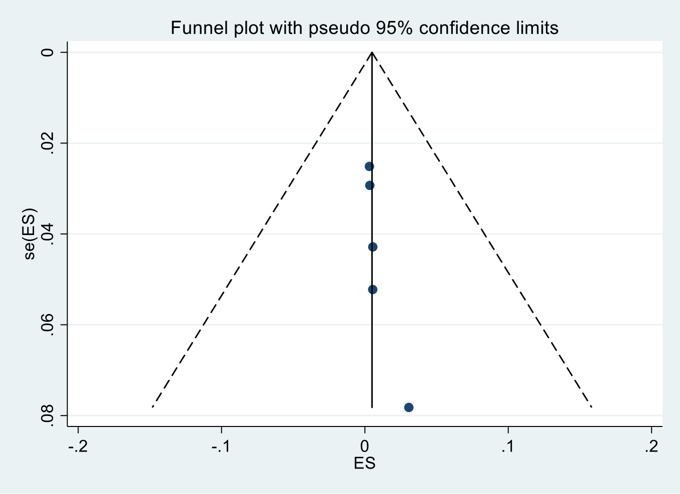	0.081

Type 1 gNET	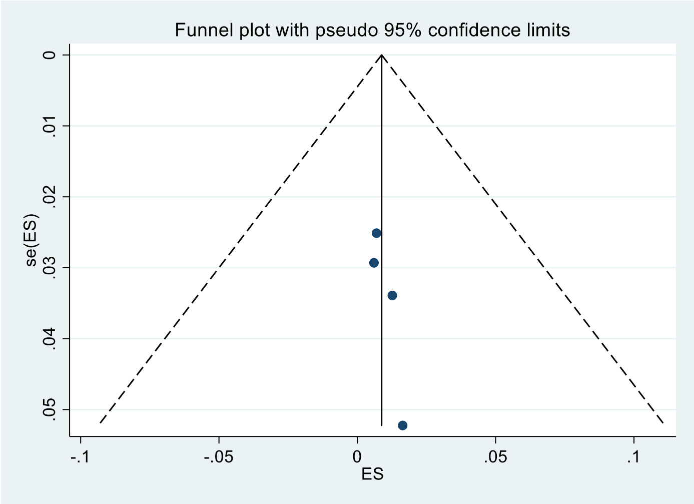	0.134

Figure S1. Sensitivity analysis [9–11,13–16,18]

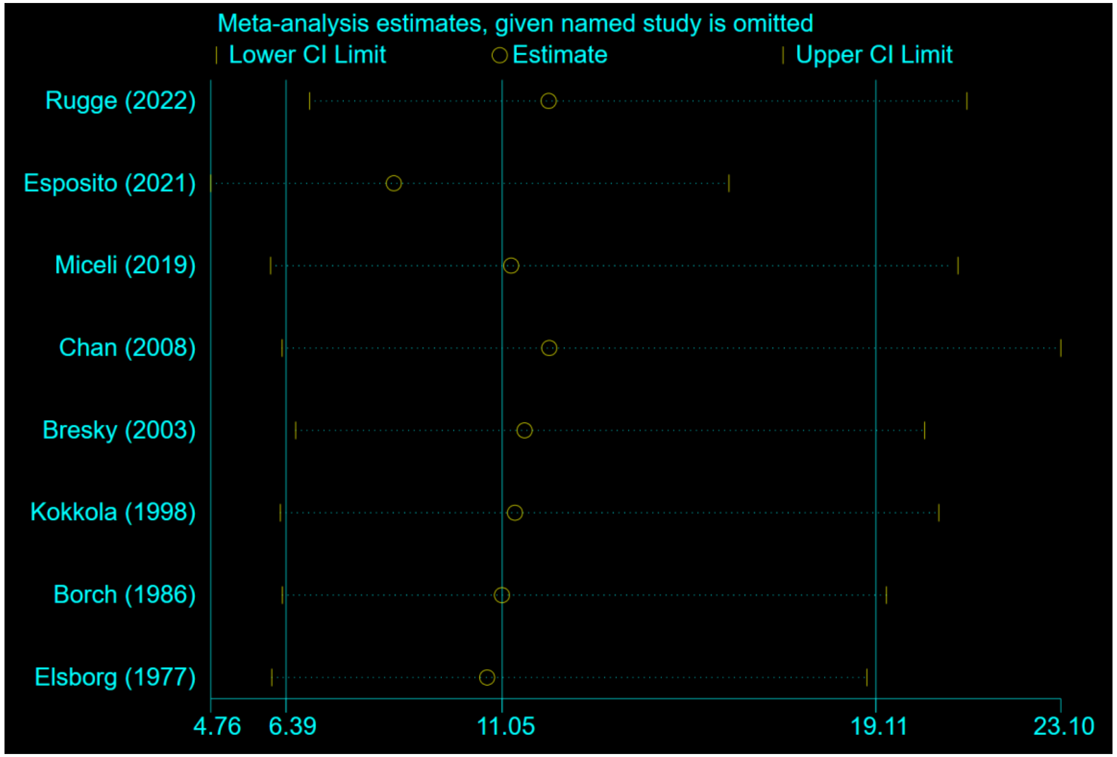

Supplement: Supplementary file 1 [file jcm-12-01062-s001.zip › jcm-2107497-supplementary.pdf]
